# Generalizing from qualitative data: a case example using critical realist thematic analysis and mechanism mapping to evaluate a community health worker-led screening program in India

**DOI:** 10.1186/s13012-024-01407-2

**Published:** 2024-12-24

**Authors:** Kathryn Broderick, Arthi Vaidyanathan, Matthew Ponticiello, Misha Hooda, Vaishali Kulkarni, Andrea Chalem, Puja Chebrolu, Ashlesha Onawale, Ana Baumann, Jyoti Mathad, Radhika Sundararajan

**Affiliations:** 1 Department of Family Medicine, Sydney Kimmel Medical College, Philadelphia, PA USA; 2 School of Medicine, Duke University, Durham, NC USA; 3 School of Medicine, Yale University, New Haven, CT USA; 4 Center for Global Health, Weill Cornell Medicine, New York, NY USA; 5 Deep Griha Society, Pune, Maharashtra India; 6 Department of Epidemiology, Gillings School of Global Public Health, University of North Carolina, Chapel Hill, NC USA; 7 Division of Public Health Sciences, Department of Surgery, Washington University in St Louis, St Louis, MO USA; 8 Department of Medicine, Weill Cornell Medicine, New York, NY USA; 9 Department of Emergency Medicine, Weill Cornell Medicine, New York, NY USA

**Keywords:** Qualitative data, Generalizing, Mechanism mapping, Critical realism

## Abstract

**Background:**

A central goal of implementation science is to generate insights that allow evidence-based practices to be successfully applied across diverse settings. However, challenges often arise in preserving programs’ effectiveness outside the context of their intervention development. We propose that qualitative data can inform generalizability via elucidating mechanisms of an intervention. Critical realist thematic analysis provides a framework for applying qualitative data to identify causal relationships. This approach can be used to develop mechanism maps, a tool rooted in policy that has been used in health systems interventions, to explain how and why interventions work. We illustrate use of these approaches through a case example of a community health worker (CHW)-delivered gestational diabetes (GDM) screening intervention in Pune, India. CHWs successfully improved uptake of oral glucose tolerance tests (OGTT) among pregnant women, however clinical management of GDM was suboptimal.

**Methods:**

Qualitative interviews were conducted with 53 purposively sampled participants (pregnant women, CHWs, maternal health clinicians). Interview transcripts were reviewed using a critical realist thematic analysis approach to develop a coding scheme pertinent to our research questions: “What caused high uptake of GDM screening?” and “Why did most women with GDM referred to clinics did not receive evidence-based management?”. Mechanism maps were retrospectively generated using short- and long-term outcomes as fenceposts to illustrate causal pathways of the CHW–delivered program and subsequent clinical GDM management.

**Results:**

Critical realist thematic analysis generated mechanism maps showed that CHWs facilitated GDM screening uptake through affective, cognitive and logistic pathways of influence. Lack of evidence-based treatment of GDM at clinics was caused by 1) clinicians lacking time or initiative to provide GDM counseling and 2) low perceived pre-test probability of GDM in this population of women without traditional risk factors. Mechanism mapping identified areas for adaptation to improve the intervention for future iterations.

**Conclusions:**

Mechanism maps created by repeated engagement following the critical realist thematic analysis method can provide a retrospective framework to understand causal relationships between factors driving intervention successes or failures. This process, in turn, can inform the generalizability of health programs by identifying constituent factors and their interrelationships that are central to implementation.

**Supplementary Information:**

The online version contains supplementary material available at 10.1186/s13012-024-01407-2.

Contributions to the literature

- Qualitative data can be analyzed through a critical realist lens to inform understanding of causal mechanisms. Despite using hyper-local and highly contextual data, critical realist thematic analysis can be used to identify causal mechanisms to inform generalizability of interventions.
- This work demonstrates a novel application of mechanism mapping generated by using retrospective critical realist thematic analysis of qualitative data from a study in Pune, India where community health workers were trained to deliver screening for gestational diabetes in urban, slum communities.
- Qualitatively-derived mechanism maps identified areas where our hypothesized mechanisms of change did not align with observed mechanisms. This reflexive process illustrates the importance of using data to interrogate assumptions driving initial logic model development.
- Discordance between expected and observed outcomes an also guided program adaptation for our ongoing clinical trial. Our use of critical realist thematic analysis can contribute to implementation generalizability by illustrating a process that could be replicated to adapt evidence-based programs for delivery at a larger scale or in different contexts.

## Introduction

Critical realism is a philosophical approach that has become increasingly applied to the field of implementation science to explain the process and outcomes of implementation. The strength of critical realism in implementation science is that it accounts for the complex nature of evidence-based interventions and focuses on explaining what works under specific conditions and contexts [1]. A critical realist lens interrogates the relationships between individuals and their contexts [2, 3], as well as influences by structures and other agents, to identify causal mechanisms and their effects on health outcomes. The critical realist approach conceives of structures and conditions in specific contexts acting through mechanisms to produce the observed effect or event [4]. While mechanisms are typically not directly observable, they are identified through the process of *retroduction* [5] – working backwards from empirical events and identifying causal forces that explain the events observed. Through this iterative process, researchers propose multiple explanations (or mechanisms) and investigate their validity through data corroboration [6].

Identifying mechanisms to understand causation within an implementing system can potentially inform generalizability of evidence-based programs, a central aspect of facilitating effective and equitable delivery of evidence-based care [7–9]. Generalizability refers to the applicability of findings to an unknown or wider population and is central to the implementation science mission of reducing the evidence-practice gap. To facilitate maximum impact of testing interventions in a specific study context, it is critical to understand the causal mechanisms at play during implementation.

An existing approach in implementation science to identify and evaluate mechanisms empirically is using quantitative data to create directed acyclic graphs (DAGs) [10–12], which allows for weighted modeling of mechanisms. A weakness of using hypothetical DAGs, however, is that they are limited in scope to variables known to the researcher. Novel variables are challenging to capture using these methods as the variables are, by definition, generated by the researcher. Qualitative data, in contrast, allows for novel variable capture by including the voices of study participants. Realist evaluations in implementation science have drawn from theories of critical realism to understand how and why interventions work under different circumstances [13]. Using a “context + mechanism = outcome” formula as a guiding principle, realist studies focus on linking contextual drivers with clinical outcomes through theoretical mechanisms of action. One challenge with realist approaches has been a paucity of standardized methods or protocols for this type of analysis, leading to varied analytical methods and confusion among researchers [14]. Pawson & Tilley recommend using mixed methods data to evaluate hypothesized context-mechanism-outcome relationships [15]. However, realist evaluations do not routinely engage with these data to identify mechanisms, with a few notable exceptions [16–18].

Fryer (2022) posits that thematic analysis – a method common to qualitative research – should be operationalized under the lens of critical realism to “produce nuanced causal explanations of events, countering the mistaken assumption that qualitative research cannot produce causal knowledge” [19]. Qualitative data provide critical details on the implementation context of an intervention, the possibility for inductive knowledge generation, and describes structural and interpersonal parameters that influence measured (as well as unexpected) study outcomes. These data, therefore, are uniquely positioned to theorize detailed mechanisms, which can be applied to the task of creating generalizable knowledge about implementation. Despite the strength of qualitative data to speak to these issues, very few researchers have engaged with critical realist approaches to qualitative data in this way. Here, we embrace Fryer’s assertion that highly contextual qualitative data can be leveraged to elucidate detailed understandings of causal relationships between implementation structures, context, and outcomes.

In this paper, we show how critical realist thematic analysis can be utilized to provide a deeper understanding of intervention generalizability through identifying causal mechanisms to generate mechanism maps. Mechanism mapping, which has previously been used to understand outcomes in health systems interventions [20, 21], breaks down an intervention into its constituent parts, relating components of implementation strategies and how these influence intervention outcomes [22]. Mechanism maps expand upon the structure-context-mechanism-outcome framework in critical realism to include non-linear and intertwined relationships of an implementation strategy, the core steps of an intervention, how these pathways interact with each other, and how contextual factors influence one or more mechanisms [21]. Mechanism mapping models intend to explain how a proposed intervention’s theory of change interacts with its context, providing a systematic, data-driven approach to identifying the causal mechanisms driving intervention outcomes [23]. To illustrate this, we provide an example using data from a maternal health intervention in Pune, India where community health workers (CHW) were trained to deliver home-based gestational diabetes (GDM) screening in two slum communities. Through this example, we illustrate how a retrospective critical realist thematic analysis approach was used to create mechanism maps that, in turn, can inform next steps in scaling up this intervention.

### Overview of CHW-delivered gestational diabetes study

We conducted an explanatory mixed methods study of gestational diabetes screening in Pune, India, This study was conducted from October 2021 to June 2022 [24]. The work was conducted in collaboration with the Deep Griha Society, a local non-governmental organization with nearly 50 years’ experience providing child welfare, nutrition support, and health promotion programs in Pune’s slum communities (https://deepgriha.org). Details of the intervention are described in Chebrolu et al., 2023 [24]. In brief, we trained five community health workers (CHWs) from Deep Griha Society to conduct oral glucose tolerance tests (OGTTs), the gold standard screening tool for gestational diabetes, in people’s homes. We recruited 248 pregnant women in our study; of these 90% (*n* = 223) accepted the OGTT delivered by the CHW. Thirty-one women (14%) screened positive for GDM and were referred to antenatal clinics for GDM care by the CHWs. After two weeks, CHWs followed up in person with the women that screened positive for GDM to determine if they had sought care and to ask about clinical management, if any. Nearly all women with GDM had sought clinical care (97%, *n* = 30); however, only 33% of these (*n* = 10) received any counseling or treatment from the clinician for GDM. No incentives were provided to pregnant women to encourage acceptance of the OGTT or to attend clinic following screening.

At study completion, a female Ph.D. social scientist not affiliated with the study conducted 53 semi-structured interviews in Marathi (the local language) with a purposive sampled subset of 30 pregnant women (20 of whom had screened positive for GDM), all 5 CHWs, and 18 maternal health clinicians from the Pune area. Participants were identified to represent those with and without GDM; those who screened positive for GDM were purposefully overrepresented so that we could learn about the continuum of care after clinic referral, as well as clinicians from both public and private facilities providing antenatal care in the study region. Characteristics of participants in the qualitative study are shown in Appendix Table 3. Interview sample size was determined based data saturation estimates in the literature and confirmed through data analysis [25]. Qualitative interview participants received a gift (either household staples or snacks) valued at approximately 200 rupees (approximately $2.50 US Dollars).

An interview guide was developed based on the Consolidated Framework for Implementation Research framework and used to ensure consistency of topics across interviews while allowing for novel concepts to arise (S1- S3 Text). Pregnant women were asked about receiving screening from CHWs and experience with clinical GDM care. CHWs were asked about providing GDM screening and counseling in their communities. Clinicians were asked about their perceptions of GDM prevalence, diagnosis, and treatment. Interviews were audio recorded, transcribed, and translated from Marathi into English for analysis by a professional translation service. Both Marathi and English transcripts were produced for each interview. One quarter of all English transcripts were spot-checked against the Marathi transcripts and discussed with the study’s qualitative interviewer – who is fluent in both Marathi and English – to ensure preservation and fidelity of meaning.

We retroductively analyzed these qualitative data using critical realist thematic analysis to empirically create mechanism maps explaining the causal relationships between context, implementation strategies, and observed clinical outcomes. The process of the critical realist thematic analysis and mechanism map creation is presented here as an example to illustrate application of this novel approach.

## Methods

### Logic model creation

**Fig. 1:**
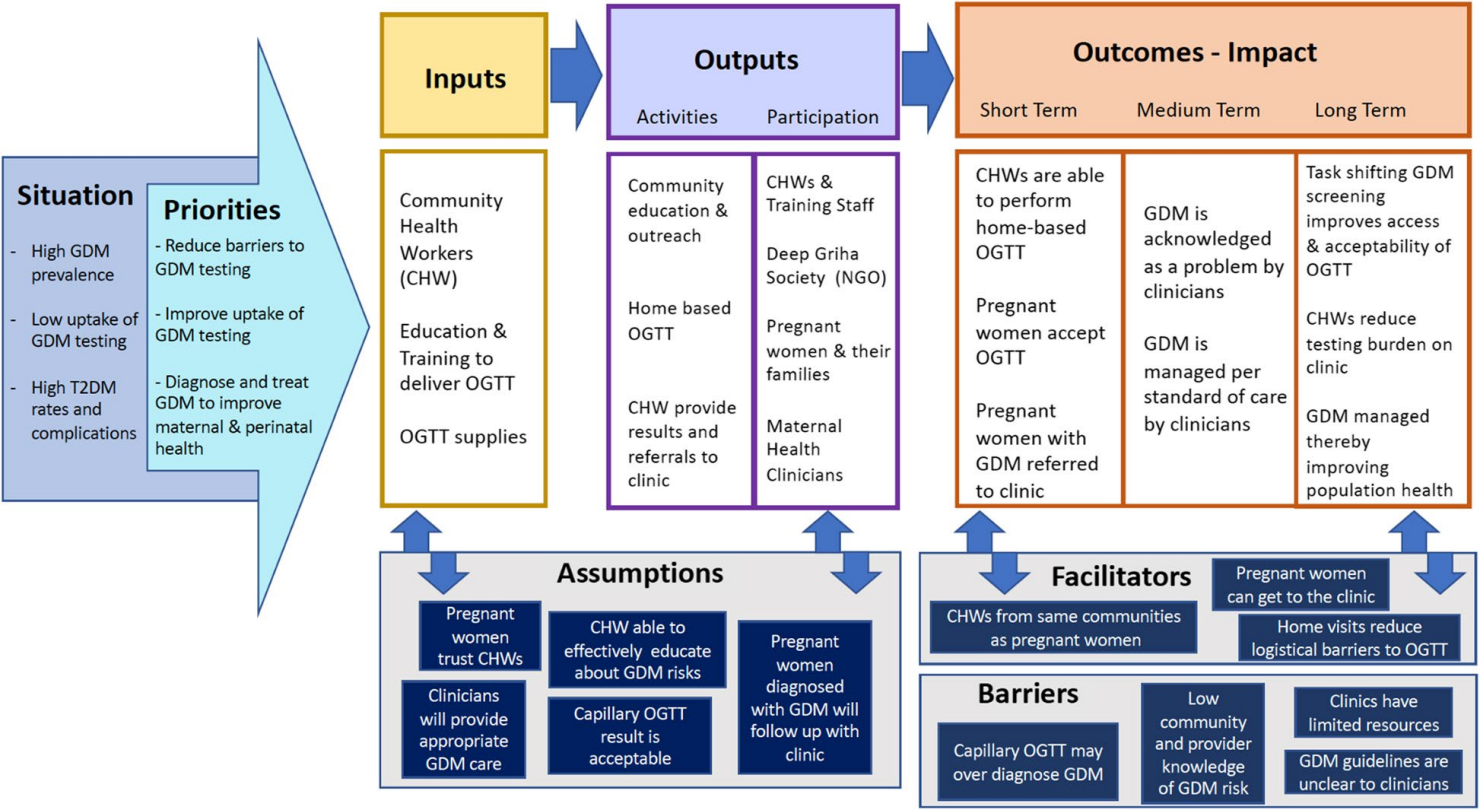
Logic model summarizing the proposed theory of change of CHW-facilitated GDM screening

Prior to study initiation, a logic model describing the intervention’s theory of change and proposed facilitators and barriers was created following repeated engagement with the literature on CHW task-shifting interventions, the study team’s prior experience with maternal health and community-based research, and informal conversations with colleagues and community health workers [26]. This logic model summarizes, in broad strokes, the anticipated process, facilitators, barriers, and outcomes of the intervention (Fig. 1). With OGTT administration training and material support, we anticipated that the CHWs would successfully engage in community outreach, home-based testing, and referrals to clinical care. The intended outcomes of this process were uptake of the OGTT among pregnant women and referral to clinical care for those screening positive for GDM (short term), individual GDM management (medium term), and improved population maternal health (long term).

Our quantitative data demonstrated that our CHW-delivered screening program resulted in achievement of anticipated short term clinical outcomes but did not align with the hypothesized medium–term outcome of women receiving evidence-based GDM management, and therefore would preclude achievement of the long-term goal of improved population maternal health. This prompted us to critically re-evaluate our qualitative data with a focus on explaining why our program failed to achieve distal outcomes, a “pre-mortem” approach that has been suggested to leverage hindsight to generate an explanation and prevent a poor outcome in the future [27].

## Qualitative data analysis

All steps in our retrospective analysis process are summarized in Table 1 and discussed in detail below.

**Table 1:** Summary of mechanism map creation

*Phases*	*Process*
Logic Model creation	1. Created implementation logic model based on the proposed theory of change integrating literature, study team experience, and contextual knowledge as appropriate 2. Predefined short-, medium-, and long-term anticipated intervention outcomes
Critical realist thematic analysis [19]	1. Study team retrospectively defined events and research questions of interest 2. Two coders familiarized themselves with the data and generated data-led descriptive codes 3. Two coders applied standardization and consolidation to develop themes (i.e., mechanisms: causal explanations of experiences/events), which were iteratively refined by four authors familiar with the data set 4. Themes were tested for validity through corroboration and re-engagement with primary data 5. Mechanism maps were drafted then interrogated for validity by the entire study team, with reversion to steps 3&4 above in cases of disagreement
Map creation	1. Short-, medium- and long-term outcomes from implementation logic model were used as fenceposts of mechanism maps 2. Contextual relationships identified by thematic analysis were placed between appropriate fenceposts 3. Codes from qualitative data identified contextual factors that influence linkage of steps and organize map into pathways 4. Arrows were placed to represent the directionality of causal relationships 5. Reengaged with qualitative data to consider additional influences between identified steps across pathways, with reversion to steps 2–4 above as needed
Concordance or discordance of observed versus predicted outcomes	1. Compared implementation logic model (created from engagement with prior literature) with mechanism maps (created from critical realist analysis of qualitative data), and identified stage/s where implementation did not proceed as expected 2. Reengaged with thematic analysis codes to identify the contextual factors that drove the deviation from our a priori theory of change 3. A. When outcome was not expected: Study team discussed actions that would potentially modify the context leading to the desired implementation outcome for future work 4. When mechanism was not expected: Study team considered how the implementation could facilitate replication of the unexpected mechanisms to improve generalizability of findings

### Critical realist thematic analysis

Interview transcripts were reviewed, using a critical realist thematic analysis approach described by Fryer [19], to develop a coding scheme pertinent to our research questions: “What caused high uptake of the CHW-delivered GDM screening?” and “Why did most women GDM referred from this study not receive evidence-based treatment at the clinics?”.

We followed Fryer’s approach to critical realist thematic analysis as follows:

- Step 1: We began by establishing our events (the experience of CHW-delivered GDM screening) and defining our two research questions, as above.
- Step 2: Authors KB, MP, and AC familiarized themselves with the qualitative data, skimming a large portion of the interviews and taking general notes. Initial descriptive codes were then generated using a data-led approach. Identified codes and themes were organized using Microsoft Word and Excel. Pertinent quotes were highlighted in Word then copied and pasted into Excel. Each participant ID was placed in an Excel row and initial descriptive codes placed in separate columns. Illustrative quotes were placed to provide “evidence” for the descriptive codes.
- Step 3: Authors KB, MP, and AC reviewed codes to ensure standardization (using the same word for similar codes) and facilitate consolidation (bringing similar thematic codes together into themes). Disagreements or discrepancies in codes were resolved through discussion between the three authors and senior author RS. An updated Excel sheet was created with consolidated codes explaning the two research questions: 1) high uptake of the CHW-delivered screening and 2) paucity of evidence-based treatment for women with GDM referred to local clinics.
- Step 4: Causal explanations were drafted from these revised codes and iteratively tested for validity by re-reading the interviews in full through the lens of the themes to test for validity. While Fryer describes causal explanations as “themes”, we present them here as “mechanisms” insofar as these are factors we identified as influencing outcomes within the study context.
- Step 5: Graphs of causal mechanisms and their relationships to intervention context/outcomes were created and then reviewed across the authorship team to interrogate the validity of the conclusions made. For cases where the authorship team did not agree on causal mechanisms or the map components, we reverted to Steps 3 and 4 again to review the primary data. After mechanisms and maps were finalized through consensus, we initiated discussions on how best to disseminate these findings and agreed on creating this methodology paper to report our process and results.

### Mechanism map creation

**Fig. 2:**
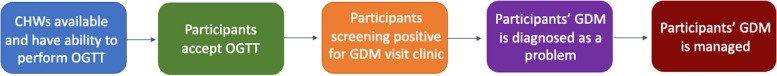
Anticipated short- and medium-term outcomes serve as fenceposts for the intervention’s mechanism map

Drafts of the mechanism maps were created by authors KB and AV based on the ‘themes’ identified during analysis to illustrate the linkage of component steps to mechanisms within the study context [28]. The intervention’s short- and medium-term outcomes – identified by the initial logic model – were placed as fenceposts for the maps (Fig. 2). Mechanism maps were then generated by positioning the key steps in relation to one another and connecting the identified causal relationships as identified by critical realist thematic analysis. Repeated engagement with the data throughout the process ensured that maps reflected participant experiences [29]. Representative quotes supporting the development of the mechanistic pathways can be found in Appendix Tables 4 and 5.

### Discordance of observed outcomes with logic model

In cases where the outcomes predicted by our implementation logic model were not observed, mechanism mapping interrogated the gap between initial and empirically derived mechanisms. First, authors KB and AV revisited the logic model and utilized the thematic analysis to identify which stage(s) the intervention failed to align (Appendix Figure 5). Authors then reengaged with qualitative analysis to characterize the contextual factors that precipitated the observed breakdown and to generate ideas regarding context modifications that would bridge the implementation gap. This included consideration of outcomes that aligned with the logic model but worked through unanticipated mechanisms. This learning intends to target improvement of the intervention to more effectively achieve long-term goals of improving population health.

## Case example

### Research question 1: What caused high uptake of the CHW-delivered GDM screening?

**Fig. 3:**
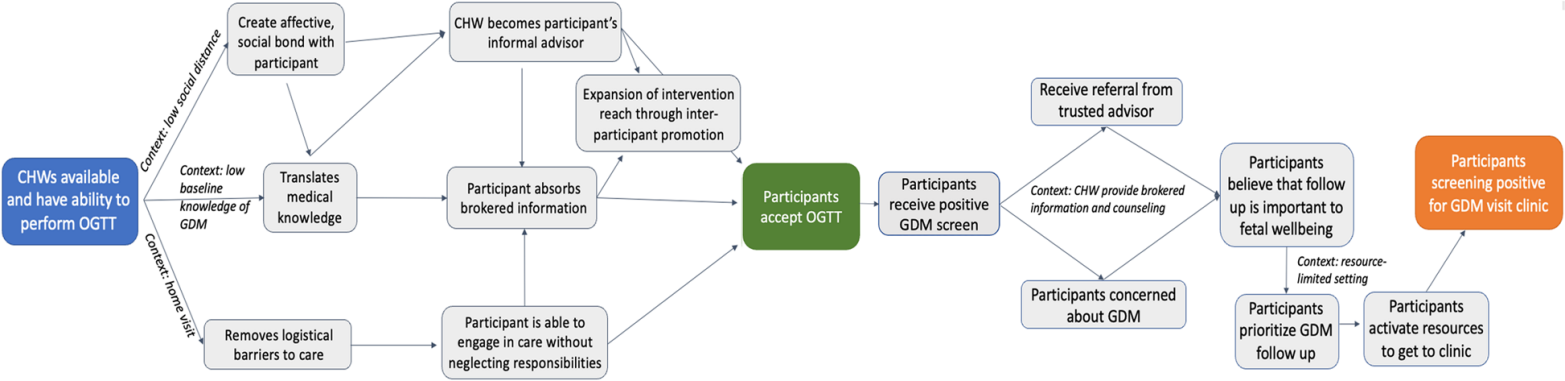
Mechanism map explaining high OGTT uptake and high rates of presentation to clinic for GDM care

CHWs trained in the delivery of home-based OGTT successfully improved GDM screening through three distinct mechanisms: affective, cognitive, and logistic influences (Fig. 3). CHWs operated affectively in the context of low social distance, as peers and members of the same community, which facilitated the formation of social bonds and allowed them to serve in the role of informal advisors. Pregnant participants described looking to CHWs for guidance on health-related topics and stated that CHWs explained the OGTT and GDM in a manner they understood, given low baseline knowledge on GDM in the communities [30, 31]. Lastly, participants cited the need for transportation and time away from the household as barriers to clinic based GDM screening care.

CHW-delivered counseling resulted in nearly all participants who screened positive for GDM presenting to clinic within two weeks. The context of CHW-provided brokered information and counseling served as an important backdrop. Affectively, the social bond between the CHW and the participant was important because the recommendation to seek a higher level of care came from a trusted peer. Cognitively, participants’ new understanding of their risk of GDM led to concern for her fetus. The confluence of both affective and cognitive factors led to the prioritization of attending clinic despite the aforementioned barriers in this low-resource setting.

### Research question 2: Why did most women with GDM referred from this study not receive evidence-based treatment at the clinics?

Once pregnant women who screened positive for GDM presented to clinic, interviews indicated numerous drivers of inconsistent management by maternal health clinicians. The standard of care involves a fasting plasma glucose OGTT to confirm the diagnosis of GDM [32]. Clinician participants, however, expressed skepticism regarding the validity of our CHW-delivered GDM screening test and were uncertain or unaware of a protocol to repeat an OGTT with a venous blood draw. Clinicians also had low concern for GDM in this low-income population, given their lack of traditional risk factors such as obesity, thereby reducing their pre-test probability that our CHW-delivered tests were accurate.

**Fig. 4:**
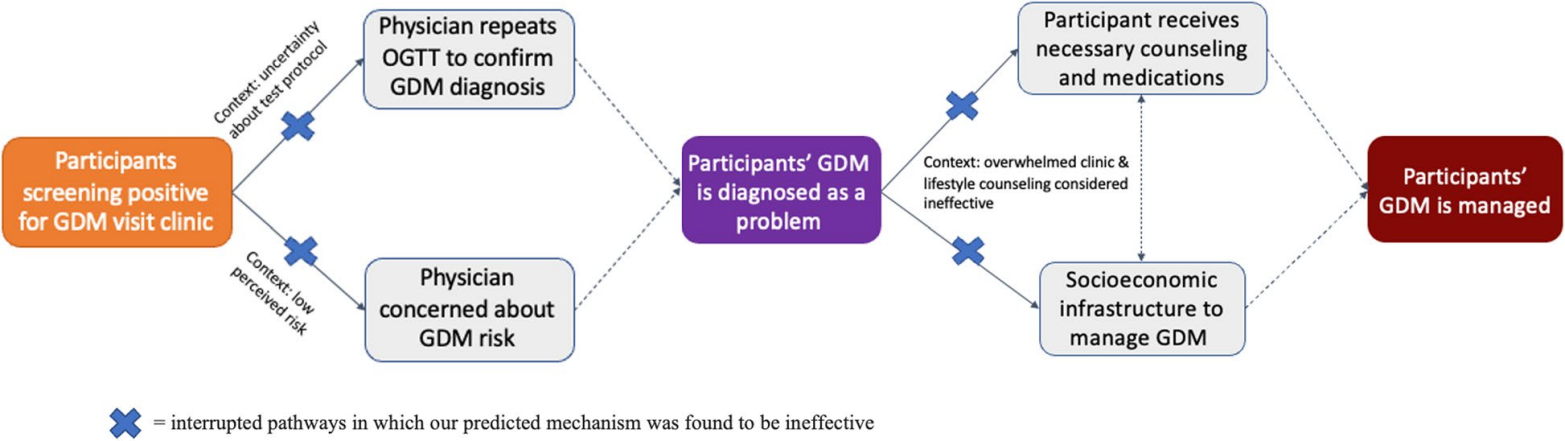
Mechanism map of clinical management for participants screening positive for GDM

Very few women who screened positive for GDM by the CHW-delivered test were provided with any recommendations regarding the need for medication or dietary changes. Some clinicians expressed concern that individual counseling would be ineffective in the cultural and socioeconomic context of slum communities, citing beliefs that poor pregnant women were active and did not have access to unhealthy foods – and therefore were not at risk for GDM. Moreover, clinicians operated within the context of *temporal scarcity*, operating above capacity and without adequate staffing, so some stated they did not have the time to provide lifestyle or nutritional counseling (Fig. 4).

## Discussion

Mechanism mapping using qualitative data through a critical realist lens provided insight into the causal relationships driving the observed outcomes of our CHW-delivered GDM screening program. To our knowledge, this is the first study to retrospectively create mechanism maps to understand drivers of implementation using critical realist thematic analysis of qualitative data. We posit that qualitative data can be used to identify mechanistic relationships explaining intervention outcomes, brings to the forefront important considerations for generalizability of findings. Our work also addresses the call to interrogate challenges in implementation science research [27, 33]; the mismatch between the assumptions in our original logic model and observed clinical outcomes was the nidus from which this reflexive engagement grew.

Generalizability is a central component of scalability [34] – continuously increasing reach or adoption of an intervention across populations. We believe that the methodological approach illustrated here can facilitate progress towards study generalizability by illustrating determinants of implementation. In their consideration of ‘scaling out’ of evidence-based programs, Aarons et al. (2017) state implementation scientists must determine if “there is sufficient empirical evidence or justification that this evidence-based program would impact health as expected” in a new context [35]. Our use of critical realist thematic analysis and mechanism mapping can provide an evidence base for generalization, by explaining *how* the program worked (and did not work) and illustrating the core mechanisms contributing to desired clinical outcomes of screening uptake and evidence based GDM management. Further, the task of generalizability is strongly linked to a thorough understanding of context [36]. Our approach accounted for the influence of highly detailed, local experiences on study outcomes while extrapolating on causality in overarching way that can be used to frame evaluations of other study settings. Our approach to mechanism mapping informs generalizability of implementation research by methodically evaluating the intervention and teasing apart what aspects of intervention success or failure could be modifiable and which were inextricably tied to contextual factors. We interrogated our original theory of change, organizing data to elucidate the steps and contextual elements that contributed to the actual implementation outcomes. The retroductive observation of mechanisms from the data by building a mechanism map allowed for reflection on the assumptions underlying our theory of change. In turn, the comparison between theoretical and indirectly observed mechanisms facilitated changes to the intervention to better match the causal mechanisms found through this critical realist analysis. This approach aligns with the “bottom up” strategy of generalization described by Borgstede & Scholz (2021) [37].

Unfortunately, our initial logic model was misaligned with the implementation context, a circumstance that has been described in other study circumstances [20, 21, 27], highlighting the need for rigorous and thorough pre-implementation work to identify these types of barriers. Our findings also demonstrate the importance of bringing empirical data to challenge and potentially deconstruct errors in researchers’ assumptions. Reflexivity of the researcher is essential to advancing an equitable approach to implementation science [38], and a central component of reflexivity is laying bare one’s assumptions. Reflexivity is even more critical when conducting global health research where health systems are already vulnerable and under-resourced, and pragmatic research – such as ours – places additional strain on these systems [39]. Our critical realist thematic analysis and mechanism maps demonstrated the need to support clinicians caring for women with GDM, which we have integrated into a central pillar of the ongoing cluster-randomized clinical trial which expands upon our pilot work (NCT06209411).

Mechanism mapping done in this manner can also unveil the relative importance of correctly predicted mechanisms. For example, based on our engagement with prior data, we believed that reducing logistical barriers was the primary benefit of this community-based screening program; however, our data showed that CHWs’ peer advisory roles was a major driver of OGTT uptake and presentation to clinical care for women screening positive for GDM. Finding this affective pathway can inform generalizability and transferability. Table 2 summarizes the mechanisms of action in identified in our analysis, and contextual considerations regarding generalization to other settings. These considerations of how mechanisms affect implementation outcomes are not limited to CHW-delivered or community-based programming. For example, our study notes that activating social networks through near-peer relationships between CHWs and women in this study was an important mechanism facilitating uptake of evidence-based GDM screening. Peer-delivered counseling may facilitate adoption by activating the affective mechanism more successfully than nurse- or physician-delivered programs. This is borne out in the literature, where peer support has been described as a highly effective approach in both community- [40–42] and facility-based [43, 44] interventions.

**Table 2:** Summary of observed mechanisms and contextual considerations for generalizing to other contexts

*Identified mechanism of action*	*Contextual considerations for this activating mechanism in other settings*
Affective pathway: Low social distance (i.e., near peer relationships) facilitates successful implementation	• Leveraging social capital and trust to increase adoption may be accomplished through training near-peer health workers such as CHWs • Programs using more specialized health worker cadres (such as nurses or social workers) may not activate the affective pathway due to greater social distances • Programs using CHWs from outside of the local community may not activate the affective pathway as effectively • Partnerships with well-regarded community-based organizations can build trust in the evidence-based program
Cognitive pathway: Knowledge brokering (i.e., translating health information) facilitates successful implementation	• Health workers can facilitate engagement with evidence-based health programs and services by ‘translating’ health information into local vernacular • Health workers/counselors that use specialized terminology may be less effective in activating the cognitive pathway through knowledge brokering
Logistic pathway: Removing logistical barriers to evidence-based programs can facilitate successful implementation	• Home-based delivery may have the greatest impact in contexts where individuals experience significant logistical barriers to care such as due to poverty, geographic marginalization, competing priorities • Reducing logistic barriers through decentralizing access to services can be particularly impactful in contexts where health systems are overburdened • Contexts where individuals have higher self-efficacy in navigating health system logistics may not benefit from activating this mechanism, as some may prefer receiving facility-based services
Clinician knowledge: Clinician knowledge of evidence-based practices can facilitate or hinder delivery of gold-standard care	• In contexts where clinician knowledge of evidence-based practices is highly variable, implementation could be optimized by determining current practices and potentially engaging clinicians in intervention planning • In contexts where clinician knowledge or buy-in is low, fidelity of delivering evidence-based practices may be strengthened through clinical champions, and/or targeted quality-improvement strategies such as coaching, audit/feedback, educational programming, etc. • Decentralized screening programs can improve uptake of screening, but its impact is limited without gold standard clinical care for individuals who require facility-based treatment
Temporal scarcity: Implementation of evidence-based care is challenging in resource-limited settings	• Clinicians may not prioritize delivering evidence-based care in overburdened clinical settings if the program is perceived to be lengthy and time consuming. Less complex options may be favored in these contexts • Researchers may advocate for changes in policy and/or funding to address structural drivers of health – such as lack of clinical resources – in low-resource contexts

Using a critical realist thematic analysis approach to identify causal mechanism to explain intervention outcomes can also provide targets for adaptation. While qualitative data used to inform adaptation is highly contextual, the process of adaptation potentially increases intervention generalizability by creating interventions that are more responsive and aligned with structural and social constraints [45]. While our intervention was highly successful in its originally stated goal (GDM screening uptake), our medium-term goal of improved GDM management was not achieved due to observed clinical management that felt short of gold standard, evidence-based management. By using mapping to further understanding what those factors are and how they influenced study outcomes through qualitative data, adaptations can be designed overcome barriers or replicate facilitators to foster intervention success in more generalized settings. By disaggregating the intervention into component, related parts, our analysis pinpointed areas for targeted adaptations to improve medium- and long- term participant outcomes in various contexts. Our illustration of targeted adaptation intends to contribute to literature on implementation generalizability by highlighting how adaptations can improve engagement as well as clinical effectiveness [46].

## Conclusion

Mechanism maps generated through critical realist thematic analysis of qualitative data can provide a detailed understanding of causal relationships driving implementation of evidence-based practices. Qualitatively derived mechanism maps could be used to generalize implementation of evidence-based programs across global contexts.

## Supplementary Information

Supplementary Material 1.

Supplementary Material 2.

Supplementary Material 3.

Supplementary Material 4.

## Figures and Tables

**Fig. 5:**
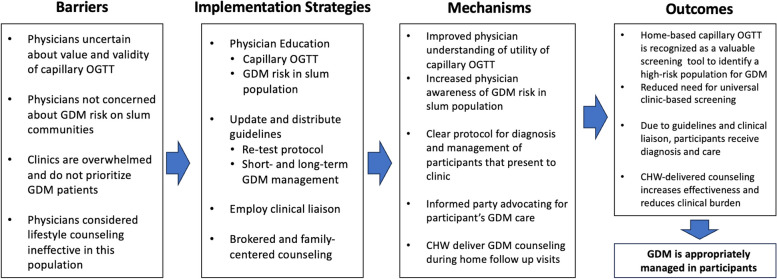
Revised logic model summarizing proposed intervention changes to address inconsistent clinical management of GDM

## Data Availability

The datasets generated and/or analysed during the current study are not publicly available but are available from the corresponding author on reasonable request. The TIDieR and COREQ reporting checklists are available as supplemental materials.

## Appendix

**Table 3:** Parent study qualitative interview participant characteristics

	**Age in years** (median [IQR])	**Gestational age at enrollment in weeks** (median [IQR])	**Marital status** (n, %)	**Religion** (n, %)	**Highest educational level** (n, %)	**Occupation** (n, %)
**All pregnant women** (*n* = 30)	23 [6]	26.1 [6.1]	Married (30, 100%)	Hindu (20, 66.7%) Muslim (5, 16.7%) Buddhist (5, 16.7%)	Graduate school (4, 13.3%) Post-high school certificate (8, 26.7%) High school (12, 40%) Middle school (4, 13.3%) Primary school (1, 3.3%) None (1, 3.3%)	Housewife (27, 90%) Other (3, 10%)
**GDM + ** (*n* = 20)	23 [7]	24.9 [3.4]	Married (19, 100%)	Hindu (12, 63.2%) Muslim (4, 21.1%) Buddhist (3, 15.8%)	Graduate school (4, 21.1%) Post high school certificate (5, 26.3%) High school (7, 36.8%) Middle school (3, 15.8%)	Housewife (18, 94.7%) Other (1, 5.3%)
**GDM –** (*n* = 10)	23 [8]	30.3 [7.1]	Married (11, 100%)	Hindu (8, 72.7%) Muslim (1, 9.1%) Buddhist (2, 18.2%)	Post high school certificate (3, 27.3%) High school (5, 45.5%) Middle school (1, 9.1%) Primary school (1, 9.1%) None (1, 9.1%)	Housewife (9, 81.8%) Other (2, 18.2%)
**CHW** (*n* = 5)	30 [4]	N/A	Married (4, 80%)	Hindu (3, 60%) Muslim (2, 40%)	Post high school certificate (1, 20%) High school (4, 80%)	N/A
**Clinicians** (*n* = 18)	37 [8]	N/A	Married (18, 89%)	Hindu (14, 78%) Muslim (22%)	Graduate school (18, 100%)	OB-GYN (15, 83%) General Practitioner (3, 17%)

**Table 4:** Representative quotes illustrating mechanisms of OGTT uptake and clinic presentation

Mechanism map pathway	Qualitative finding	Representative Quotes
**CHWs available and have ability to perform OGTT** **Participants accept OGTT**	Low social distance creates affective social bond, CHW as informal advisor	"She talks very nicely. It didn’t feel, like someone from outside is coming and doing my check-up" – Pregnant woman, 24 years old
		"When you mentioned the name *Deep Griha,* anyone would be ready. Because in every one of their homes whether it may be grandmother or grandfather, someone or the other… they know Deep Griha”- CHW, 36 years old
	CHW translates medical knowledge, patient absorbs information	"I previously did [an OGTT] at [the government hospital], but they didn't tell me anything much at all. Not like the way that [the CHW] had told me. ‘It is like this. There can be a problem.’" – Pregnant woman, 24 years old
		"They only know that they have to go to the hospital and after going there they give a bottle filled with sugar water, then they have to drink it and do the test for sugar. But why is it done, the importance of that they don’t know yet. So, when we were doing the study, we were sitting down with each of the women and their family members in their homes and explaining its importance.” – CHW, 47 years old
	CHW-delivered testing removed logistic barriers, patient has ability engage in care	“There is a large family in houses [and lots of responsibilities], so we cannot get out of the house [to do the OGTT testing].” – Pregnant woman, 25 years old
		"It saved the time. I would have spent two hours over there at the hospital, I would just have sat there. At least I did some work at home. So this was the advantage! Saved two hours …. yes, it also saved traveling time" – Pregnant woman with GDM, 22 years old
**Participants accept OGTT** **Participants screening positive for GDM visit clinic**	Participants trust CHW advice to seek follow up	**"**Since I came to know [about my GDM from the CHW] now I can take some good decisions about what I want to do going ahead."- Pregnant woman with GDM, 33 years old
		"Since we came to know [about the GDM from the CHW] we can control it…So instead of going into a depression, I can think how we can improve it. [My CHW] and the others from the Deep Griha working on providing that knowledge." – Pregnant woman with GDM, 33 years old
	Participants concerned about GDM for fetal wellbeing	"For the baby, they are willing to do everything. They will cut down on their sugar, change their diet, and do everything." – Physician, female, 30 years of experience
		“What will happen? Such a small baby … You are worried [when you have GDM]” – Pregnant woman with GDM, 30 years old

**Table 5:** Representative quotes illustrating mechanisms of inconsistent clinical management of GDM

Mechanism map pathway	Qualitative finding	Representative Quotes
**Participants with GDM visit clinic** **Participant’s GDM is diagnosed**	Physician does not trust CHW-delivered OGTT test results from capillary blood samples	"There are some variations. The standard books that we have, in them they have given the levels only for venous blood sample."—Physician, female, 11 years of experience
		“The glucometer has a ± 10 ml gram sugar of deflection compared to the standard laboratory method.” – Medical Officer, male, 20 years of experience
	Physicians not concerned about GDM risk in slum communities	"There are very few [cases of GDM] from slum areas because of their diet and lifestyle."—Physician, female, 37 years of experience
		"[GDM] is under control. It is around 5–8 percent incidence there." – Physician, female, 6 years of experience
**Participant’s GDM is diagnosed** **Participant’s GDM is managed**	Maternal health clinics are overwhelmed	"In [the government hospital], they have a heavy workload. Maybe because of that they don’t speak with the patients at all.”—Physician, male, 22 years of experience
		"[Physicians] don’t pay that much attention because there are a lot of patients there. They are in a hurry. So … they only tell you anything if you ask them. They don’t suggest anything on their own." – Pregnant woman, 20 years old
	Counseling considered ineffective in slum populations	"I mean even after telling them many times … ‘do not eat anything in between meals’, the patients don’t follow those instructions."—Physician, female, 7 years of experience
		"We advise patients every time [about diet and exercise], but patients don’t follow-up.” – Physician, female, 1 year of experience

## References

[1] Williams L, Rycroft-Malone J, Burton CR. Bringing critical realism to nursing practice: Roy Bhaskar'scontribution. Nurs Philos. 2017;18(2). 10.1111/nup.12130. 10.1111/nup.12130 27381640

[2] Bhaskar, R. A Realist Theory of Science (1^st^ ed.). London: Routledge; 2008.

[3] Bunge M. How Does It Work?: The Search for Explanatory Mechanisms. Philos Soc Sci. 2004;34(2):182–210.

[4] Sayer A. Realism and Social Science. Sage Publications; 1999

[5] Mingers J. Real-izing information systems: critical realism as an underpinning philosophy for information systems. Inf Organ. 2004;14(2):87–103.

[6] Wynn Jr D, Williams CK. Principles for conducting critical realist case study research in information systems. MIS quarterly. 2012:787-810.

[7] Lewis CC, Klasnja P, Powell BJ, Lyon AR, Tuzzio L, Jones S, Walsh-Bailey C, Weiner B. From Classification to Causality: Advancing Understanding of Mechanisms of Change in Implementation Science. Front Public Health. 2018;7(6):136. 10.3389/fpubh.2018.00136.PMID:29868544;PMCID:PMC5949843. 10.3389/fpubh.2018.00136 PMC5949843 29868544

[8] Lewis CC, Powell BJ, Brewer SK, Nguyen AM, Schriger SH, Vejnoska SF, Walsh-Bailey C, Aarons GA, Beidas RS, Lyon AR, Weiner B, Williams N, Mittman B. Advancing mechanisms of implementation to accelerate sustainable evidence-based practice integration: protocol for generating a research agenda. BMJ Open. 2021;11(10):e053474. 10.1136/bmjopen-2021-053474.PMID:34663668;PMCID:PMC8524292. 34663668 10.1136/bmjopen-2021-053474 PMC8524292

[9] Lewis CC, Frank HE, Cruden G, Kim B, Stahmer AC, Lyon AR, Albers B, Aarons GA, Beidas RS, Mittman BS, Weiner BJ, Williams NJ, Powell BJ, MNoE Group. A research agenda to advance the study of implementation mechanisms. Implement Sci Commun. 2024;5(1):98. 10.1186/s43058-024-00633-5. PMID: 39285504; PMCID: PMC11403843. 39285504 10.1186/s43058-024-00633-5 PMC11403843

[10] Rodrigues D, Kreif N, Lawrence-Jones A, Barahona M, Mayer E. Reflection on modern methods: constructing directed acyclic graphs (DAGs) with domain experts for health services research. Int J Epidemiol. 2022;51(4):1339–48. 35713577 10.1093/ije/dyac135 PMC9365627

[11] Tsai CL, Camargo CA. Methodological considerations, such as directed acyclic graphs, for studying “acute on chronic” disease epidemiology: chronic obstructive pulmonary disease example. J Clin Epidemiol. 2009;62(9):982–90. 19211222 10.1016/j.jclinepi.2008.10.005

[12] Rosenberg NE, Westreich D, Bärnighausen T, Miller WC, Behets F, Maman S, et al. Assessing the effect of HIV counselling and testing on HIV acquisition among South African youth. AIDS Lond Engl. 2013;27(17):2765–73. 10.1097/01.aids.0000432454.68357.6a PMC4028633 23887069

[13] Dalkin SM, Greenhalgh J, Jones D, Cunningham B, Lhussier M. What’s in a mechanism? Development of a key concept in realist evaluation. Implement Sci. 2015;10(1):49. 25885787 10.1186/s13012-015-0237-x PMC4408605

[14] Salter KL, Kothari A. Using realist evaluation to open the black box of knowledge translation: a state-of-the-art review. Implement Sci. 2014;9(1):115. 25190100 10.1186/s13012-014-0115-y PMC4172789

[15] Pawson R, Tilley N. Realistic Evaluation. SAGE publications; 1997.

[16] Haynes A, Gilchrist H, Oliveira JS, Tiedemann A. Using Realist Evaluation to Understand Process Outcomes in a COVID-19-Impacted Yoga Intervention Trial: A Worked Example. Int J Environ Res Public Health. 2021;18(17):9065. 34501654 10.3390/ijerph18179065 PMC8431647

[17] Tsang JY, Blakeman T, Hegarty J, Humphreys J, Harvey G. Understanding the implementation of interventions to improve the management of chronic kidney disease in primary care: a rapid realist review. Implement Sci. 2016;11(1):47. 27044401 10.1186/s13012-016-0413-7 PMC4820872

[18] Bick DE, Rycroft-Malone J, Fontenla M. A case study evaluation of implementation of a care pathway to support normal birth in one English birth centre: anticipated benefits and unintended consequences. BMC Pregnancy Childbirth. 2009;9(1):47. 19804624 10.1186/1471-2393-9-47 PMC2761848

[19] Fryer T. A critical realist approach to thematic analysis: producing causal explanations. J Crit Realism. 2022;21(4):365–84.

[20] Geng EH, Baumann AA, Powell BJ. Mechanism mapping to advance research on implementation strategies. PLoS Med. 2022;19(2):e1003918. 10.1371/journal.pmed.1003918. 10.1371/journal.pmed.1003918 PMC8824331 35134069

[21] Kilbourne AM, Geng E, Eshun-Wilson I, Sweeney S, Shelley D, Cohen DJ, et al. How does facilitation in healthcare work? Using mechanism mapping to illuminate the black box of a meta-implementation strategy. Implement Sci Commun. 2023;4(1):53. 37194084 10.1186/s43058-023-00435-1 PMC10190070

[22] Williams MJ. External Validity and Policy Adaptation: From Impact Evaluation to Policy Design. Health Nutr Popul. 2020;35(2):158–91.

[23] Williams MJ. External Validity and Policy Adaptation: A Five-step Guide to Mechanism Mapping. Policy Memo, Blavatnik School of Government, Oxford University, Oxford, UK, https://www.bsg.ox.ac.uk/research/publications/external-validity-and-policy-adaptation-0 . 2017.

[24] Chebrolu P, Chalem A, Ponticiello M, Broderick K, Vaidyanathan A, Lorenc R, et al. A community health worker-led program to improve access to gestational diabetes screening in urban slums of Pune, India: Results from a mixed methods study. PLOS Glob Public Health. 2023;3(10): e0001622. 37889879 10.1371/journal.pgph.0001622 PMC10610081

[25] Hennink MM, Kaiser BN, Marconi VC. Code Saturation Versus Meaning Saturation: How Many Interviews Are Enough? Qual Health Res. 2017;27(4):591–608. 27670770 10.1177/1049732316665344 PMC9359070

[26] Thompson HM, Clement AM, Ortiz R, Preston TM, Quantrell ALW, Enfield M, et al. Community engagement to improve access to healthcare: a comparative case study to advance implementation science for transgender health equity. Int J Equity Health. 2022;21(1):104. 35907962 10.1186/s12939-022-01702-8 PMC9339189

[27] Beidas RS, Dorsey S, Lewis CC, Lyon AR, Powell BJ, Purtle J, Saldana L, Shelton RC, Stirman SW, Lane-Fall MB. Promises and pitfalls in implementation science from the perspective of US-based researchers: learning from a pre-mortem. Implement Sci. 2022;17(1):55. 10.1186/s13012-022-01226-3.PMID:35964095;PMCID:PMC9375077. 35964095 10.1186/s13012-022-01226-3 PMC9375077

[28] Lewis CC, Klasnja P, Lyon AR, Powell BJ, Lengnick-Hall R, Buchanan G, Meza RD, Chan MC, BoyntonMH, Weiner BJ. The mechanics of implementation strategies and measures: advancing the study of implementation mechanisms. Implement Sci Commun. 2022;3(1):114. 10.1186/s43058-022-00358-3. 10.1186/s43058-022-00358-3 PMC9588220 36273224

[29] Lewis CC, Boyd MR, Walsh-Bailey C, Lyon AR, Beidas R, Mittman B, Aarons GA, Weiner BJ, Chambers DA. A systematic review of empirical studies examining mechanisms of implementation in health. Implement Sci. 2020;15(1):21. 10.1186/s13012-020-00983-3. 10.1186/s13012-020-00983-3 PMC7164241 32299461

[30] Prabh JK, Kondamuri SD, Samal S, Sen M. Knowledge of gestational diabetes mellitus among pregnant women in a semiurban hospital - A cross -sectional study. Clin Epidemiol Glob Health.2021(12). 10.1016/j.cegh.2021.100854.

[31] Shriraam V, Rani MA, Sathiyasekaran BWC, Mahadevan S. Awareness of gestational diabetes mellitus among antenatal women in a primary health center in South India. Indian J Endocrinol Metab. 2013;17(1):146–8. 23776868 10.4103/2230-8210.107861 PMC3659882

[32] International Association of Diabetes and Pregnancy Study Groups Consensus Panel; Metzger BE, Gabbe SG, Persson B, Buchanan TA, Catalano PA, Damm P, Dyer AR, Leiva Ad, Hod M, Kitzmiler JL, Lowe LP, McIntyre HD, Oats JJ, Omori Y, Schmidt MI. International association of diabetes and pregnancy study groups recommendations on the diagnosis and classification of hyperglycemia in pregnancy. Diabetes Care. 2010;33(3):676-82. 10.2337/dc09-1848. 10.2337/dc09-1848 PMC2827530 20190296

[33] Chambers DA, Emmons KM. Navigating the field of implementation science towards maturity: challenges and opportunities. Implementation Sci. 2024;19:26. 10.1186/s13012-024-01352-0. 10.1186/s13012-024-01352-0 PMC10936041 38481286

[34] Milat AJ, King L, Bauman AE, Redman S. The concept of scalability: increasing the scale and potential adoption of health promotion interventions into policy and practice. Health Promot Int. 2013;28(3):285–98. 22241853 10.1093/heapro/dar097

[35] Aarons GA, Sklar M, Mustanski B, Benbow N, Brown CH. “Scaling-out” evidence-based interventions to new populations or new health care delivery systems. Implement Sci. 2017;12(1):111. 28877746 10.1186/s13012-017-0640-6 PMC5588712

[36] Nilsen P, Bernhardsson S. Context matters in implementation science: a scoping review of determinant frameworks that describe contextual determinants for implementation outcomes. BMC Health Serv Res. 2019;19(1):189. 30909897 10.1186/s12913-019-4015-3 PMC6432749

[37] Borgstede M, Scholz M. Quantitative and Qualitative Approaches to Generalization and Replication–A Representationalist View. Front Psychol. 2021Feb;5(12):605191. 10.3389/fpsyg.2021.605191 PMC7892774 33613387

[38] Snell-Rood C, Jaramillo ET, Hamilton AB, Raskin SE, Nicosia FM, Willging C. Advancing health equity through a theoretically critical implementation science. Translational behavioral medicine. 2021;11(8):1617–25. 33904908 10.1093/tbm/ibab008 PMC8367016

[39] Adsul P, Shelton RC, Oh A, Moise N, Iwelunmor J, Griffith DM. Challenges and Opportunities for Paving the Road to Global Health Equity Through Implementation Science. Annu Rev Public Health. 2024;45(1):27–45. 10.1146/annurev-publhealth-060922-034822. (Epub 2024 Apr 3 PMID: 38166498). 38166498 10.1146/annurev-publhealth-060922-034822

[40] Wright JL, Achieng F, Tindi L, Patil M, Boga M, Kimani M, Barsosio HC, Juma D, Kiige L, Manu A, Kariuki S, Mathai M, Nabwera HM. Design and implementation of a community-based mother-to-mother peer support programme for the follow-up of low birthweight infants in rural western Kenya. Front Pediatr. 2023;3(11):1173238. 10.3389/fped.2023.1173238.PMID:37465422;PMCID:PMC10352086. 10.3389/fped.2023.1173238 PMC10352086 37465422

[41] Lu S, Leduc N, Moullec G. Type 2 diabetes peer support interventions as a complement to primary care settings in high-income nations: A scoping review. Patient Educ Couns. 2022;105(11):3267–78. 10.1016/j.pec.2022.08.010. (Epub 2022 Aug 20 PMID: 36038395). 36038395 10.1016/j.pec.2022.08.010

[42] Chang LW, Nakigozi G, Billioux VG, Gray RH, Serwadda D, Quinn TC, Wawer MJ, Bollinger RC, Reynolds SJ. Effectiveness of peer support on care engagement and preventive care intervention utilization among pre-antiretroviral therapy, HIV-infected adults in Rakai, Uganda: a randomized trial. AIDS Behav. 2015;19(10):1742–51. 10.1007/s10461-015-1159-y. 10.1007/s10461-015-1159-y PMC4567424 26271815

[43] Liebling EJ, Perez JJS, Litterer MM, Greene C. Implementing hospital-based peer recovery support services for substance use disorder. Am J Drug Alcohol Abuse. 2021;47(2):229–37. 10.1080/00952990.2020.1841218. (Epub 2020 Nov 20). 33216634 10.1080/00952990.2020.1841218

[44] White S, Foster R, Marks J, Morshead R, Goldsmith L, Barlow S, et al. The effectiveness of one-to-one peer support in mental health services: a systematic review and meta-analysis. BMC Psychiatr. 2020;20:534. 10.1186/s12888-020-02923-3. 10.1186/s12888-020-02923-3 PMC7657356 33176729

[45] Chambers DA. Advancing adaptation of evidence-based interventions through implementation science: progress and opportunities. Front Health Serv. 2023;3:1204138. 37342795 10.3389/frhs.2023.1204138 PMC10277471

[46] Lau AS, Huey SJ, Baumann AA. Advances in the adaptation and implementation of evidence-based interventions for historically marginalized groups. Behav Res Ther. 2023;168: 104377. 37531808 10.1016/j.brat.2023.104377

