# Supplementary material for: Generalizing from qualitative data: a case example using critical realist thematic analysis and mechanism mapping to evaluate a community health worker-led screening program in India
Source: Implement Sci. 2024 Dec 24;19:81. doi: 10.1186/s13012-024-01407-2 (PMC11667965; doi:10.1186/s13012-024-01407-2)
Supplement: Supplementary file 3 — Supplementary Material 3. [file 13012_2024_1407_MOESM3_ESM.docx]

**S3 Text. Interview guide for Clinicians**

1. Please tell me about your background and experience in providing medical care to pregnant women.
2. Please tell me about your experience with diagnosing and managing gestational diabetes and diabetes (Prompts: Did you learn about GDM during training/schooling? What are your thoughts on different screening methods? How do you typically screen for GDM? Is the screening process accurate/trustworthy?)
3. How do you see GDM affecting your patient community? (Prompts: do you think that GDM is common in the population that you treat? What types of pregnant women tend to develop? Do you think that GDM is dangerous? What can happen to a baby if the mother has GDM? What can happen to the mother if she has GDM?)
4. Please tell me about what would happen if a patient screened positive for GDM in your clinic. (Prompts: would they receive counseling? Would they receive medications? Would the baby receive more scans? Would you test them again after pregnancy? What would happen if they had GDM but initially tested negative for type 2 diabetes via nonfasting glucose testing?)
5. What kinds of counseling or medications do you typically provide for women who screen positive for GDM ? (Prompts: Is counseling effective? Why or why not? Are medications effective? Which ones do you tend to use and why?)
